# Fresh Ideas, Foundational Experiments (FIFE): Immunology and Diabetes 2016 FIFE Symposium

**DOI:** 10.3389/fendo.2017.00238

**Published:** 2017-09-19

**Authors:** Isobel C. Mouat, Zachary J. Morse, Virginie S. E. Jean-Baptiste, Jessica R. Allanach, Marc S. Horwitz

**Affiliations:** ^1^Department of Microbiology and Immunology, University of British Columbia, Vancouver, BC, Canada

**Keywords:** type 1 diabetes, islet encapsulation, intestinal epithelial cells, self-peptide complexes, T-cell pathogenicity, T-cell metabolism, dendritic cell activity, type 2 diabetes

## Abstract

The first Fresh Ideas, Foundational Experiments (FIFE): Immunology and Diabetes symposia workshop took place in 2016 and exemplified the active interest of a number of several investigators interested the global rise in the incidence of type 1 diabetes (T1D). This increase does not correlate with genetic drift and indicates that environmental exposures are playing an increasingly significant role. Despite major biomedical and technological advances in diagnosis and treatment, treatments are frequently insufficient as they do not inhibit the progression of the underlying autoimmune response and often fail to prevent life-threatening complications. T1D is the result of autoimmune destruction of the insulin-producing beta cells of the pancreas, and the precise, mechanistic contribution of the immune system to disease pathogenesis and progression remains to be fully characterized. Ultimately, the combinatorial effect of concurrent factors, including beta cell fragility, exogenous stressors, and genetic priming of the innate and adaptive immune system, work together to induce T1D autoimmunity. Thus, T1D is the result of immunological defects and environmental pathogens, requiring the sustained attention of collaborative research teams such as FIFE: I & D with varied perspectives, unified by the universally held goal of finding a sustainable, life-long cure. Herein, the authors provide perspective on various fields in T1D research highlighted by speakers participating in the inaugural FIFE symposium.

## Introduction

Type 1 diabetes (T1D) is an autoimmune disease of unclear etiology that results in the destruction of the insulin-producing beta cells of the pancreas, causing loss of systemic blood glucose regulation and hyperglycemia, insulin resistance and chronic joint pain (1–3). When left untreated, T1D can lead to defects in wound healing and diabetic retinopathy (4, 5), but on a daily basis, even a person with managed T1D must make significant lifestyle changes to constantly monitor blood glucose and navigate the financial burdens of insulin supplementation, medications and proper diet. Current T1D diagnostics and standard therapeutics typically address hyperglycemia without targeting the underlying autoimmune response and are thus insufficient in predicting prognosis and reducing pathogenesis long term (6–8). Damage to the pancreas is mediated by infiltrating innate and adaptive immune cells that induce pancreatic tissue pathology and disrupt molecular pathways involved in insulin regulation; however, the early events of disease initiation are still poorly understood, hindering the development of targeted immunological treatments (9–13). The steady increase in the incidence of T1D over time is not accounted for entirely by population genetics, indicating that geographic and environmental agents are important contributors to the pathogenesis of T1D (14–18). Understanding the mechanisms of the pathogenic components and their interactive roles will require the collaboration of many research teams with a variety of perspectives and approaches to tackle central questions in TID research.

Our lab focuses on researching the contribution of viral pathogens to T1D development and pathogenesis. Infection by picornaviruses such as coxsackievirus B (CVB) promotes T1D development through the stimulation of pancreatic inflammation, resulting in the release of islet antigens and the development of autoantigens. CVB antigens are recognized by pattern recognition receptors, which stimulate a type I and III interferon response (19, 20). A multifaceted immune cascade develops, characterized by the activation of innate cells, disruption of regulatory cells, and increased antigen presentation and memory cells. CVB infection is a T1D initiating influence that works in concert with other environmental factors as well as genetic variance. We aim to incorporate a perspective using models with multifactorial autoimmunity triggers by examining the contribution of gut microbiome and virome, pancreatic viral persistence, and T1D-risk genes, to determine impact on inflammation, innate sensing, and loss of regulatory mechanisms leading to onset of T1D.

As scientists work to elucidate the complex and multifactorial nature of T1D, they continue to voice a strong need for collaboration in new and innovative areas of research. With the aim to foster such collaborative endeavors, Dr. Marc Horwitz of the University of British Columbia’s Department of Microbiology and Immunology organized the first Fresh Ideas, Foundational Experiments (FIFE): Immunology and Diabetes mini-symposium, named and themed affectionately after Dr. Brian Fife, a cherished member of the JDRF nPOD network. The event centered around immunological T1D research, though this mini-symposium pushed beyond those boundaries by examining shared characteristics with multiple sclerosis (MS), defects in cellular metabolism, immunodeficiencies in type 2 diabetes (T2D), and the contribution of the microbiome to autoimmunity. A variety of approaches and experimental methods were discussed, ranging from therapeutic applications based on innovative biomaterials to translation and complementation of mouse models and clinical data for a more complete understanding of the immunological underpinnings of T1D.

## Islet Transplantation Using a Novel Encapsulation Strategy

To kick-off the symposium, Dr. Hubert Tse of the Department of Microbiology-Comprehensive Diabetes Center, School of Medicine University of Alabama, Birmingham, discussed his group’s work on engineering and applying biomaterials for effective transplantation of functional insulin-secreting beta cells in T1D patients (21). A major hindrance to successful islet transplantation is rejection due to reactive oxygen species (ROS)-induced oxidative stress and non-specific inflammation in the pancreatic microenvironment. To address this problem, the Tse lab has constructed a semipermeable, anti-oxidative material which encapsulates and protects the islets from immune destruction, while maintaining interactions between the islets and host microenvironment. The encapsulating material is formed by a layer-by-layer polymerization of anti-coagulant poly(*N*-vinylpyrrolidone) (PVPON) and the natural antioxidant tannic acid (TA), resulting in an ultra-thin, neutral and non-toxic polymeric PVPON/TA capsule (22). Preliminary *in vitro* studies using rat, non-human primate, and human islets demonstrated that PVPON/TA-encapsulated islets were able to sense glucose and secrete insulin. In addition to preserving islet function, the capsules decreased the immunoreactivity of the local microenvironment by reducing effector T-cell infiltration, chemotaxis, and synthesis of pro-inflammatory cytokines and chemokines (23). In the streptozotocin NOD.*scid* mouse model of pancreatic beta cell destruction, transplantation of PVPON/TA-encapsulated islets into the epididymal fat pad restored euglycemia as early as 2 days post-transplantation, with effects lasting up to 30 days. Hyperglycemia was then restored if the fat pads containing the transplanted islets were removed. Overall, the data demonstrate that PVPON/TA-encapsulated islets are viable, functional, and immunoprotective both *in vitro* and *in vivo*.

Rejection of allogeneic islets or pluripotent stem cells persists as the most significant challenge in transplantation treatments necessitating life-long immunosuppression which also reduces the overall functional competency of the transplanted beta cells (24). Rather than utilizing broadly acting immunosuppressive drugs, coating transplanted islets in a nano-polymer allows the islets themselves to be partially immune privileged and escape destruction from autoreactive T cells while maintaining glucoregulatory abilities. However, encapsulation strategies unfortunately also tend to limit oxygen and nutrient diffusion necessary for cell viability and also render the transplant vulnerable to cytokine-mediated toxicity and antibody recognition (25). Consequently, Dr. Tse’s work represents inventive progress in an islet transplantation packaging strategy in efforts to preserve the therapeutic effects for long-term treatment success and providing suitable islet microenvironment. However, we feel that a limitation of this project is that it does not address the inflammatory cascade induced by genetic and environmental contributors to T1D, including virus infection. Undoubtedly, treating a disease as complex as T1D will require a multifaceted approach and this research provides significant advancement in modulating local pancreatic immune responses with biomaterials in order to achieve successful islet transplantation in patients with T1D.

## Therapeutic Use of Butyrate to Alter Innate Epithelial Cell Homeostasis

To further the discussion in emerging areas of T1D research, Dr. Shannon Wallet, Associate Professor at the University of Florida’s Department of Periodontology, offered insight into the importance of the intestinal tract in development of autoimmunity. Dr. Wallet proposed that disruption of immune homeostasis in the gastrointestinal tract (GI) tract may elicit autoreactive T-cell development, activation and expansion. To examine the role of the GI tract in T1D, the Wallet group isolated and characterized immune cells in the intestinal crypts of T1D patients (26). They observed a marked expansion of the pro-inflammatory type 1 innate lymphoid cell population as well as an increase in pro-inflammatory cytokines compared to healthy controls. Dr. Wallet hypothesized that intrinsic defects in innate sensing of intestinal epithelial cells (IECs) may be responsible for the inflammation in the gut. IECs isolated from T1D patients expressed higher levels of IL-17c, an autocrine cytokine that increases pro-inflammatory responses in epithelial cells. With the aim of correcting IL-17c signaling dysregulation in the IECs of TID patient, Dr. Wallet examined the influence of administering commensal bacteria-derived butyrate to IECs as a means to promote immune regulation and suppress inflammation. *In vitro* experiments revealed that IECs from T1D patients were far less responsive to butyrate compared to controls. Specifically, butyrate was more effective at increasing the oxygen consumption rate and TSLP (thymic stromal lymphopoietin) production of IECs from control than of IECs from T1D samples. The Wallet lab is continuing to investigate the contribution of innate immune signaling and dysregulation of the GI tract on T1D and the therapeutic potential of butyrate treatment to modify pro-inflammatory IECs.

It is our perspective that an inflammatory cascade is promoted through a variety of dysregulated immune responses that interact and amplify one another. Aberrant innate sensing significantly contributes to disease through a variety of mechanisms and through multiple cell types. Innate receptors that are less experienced in some individuals due to reduced exposure to typical environmental antigens, likely cause an exaggerated or prolonged inflammatory response upon novel recognition. This prolonged inflammatory response in turn contributes to activation of autoreactive B and T cells, some with pancreatic tropism. Type 1 interferonopathies represent an example of how defects in innate sensing can lead to disease. Type 1 interferonopathies, characterized by a dysfunctional production of type 1 interferons, are often associated with autoinflammation and autoimmune phenomena (27, 28). It is the view of our lab that localized type 1 interferonopathies in pancreatic microenvironments caused by environmental, as well as genetic influences, significantly contribute to T1D (29).

Innate sensing can be altered by genetic variation, virus infections, and microbiome dysbiosis; all of which have been implicated in T1D (30, 31). Certain differences in both the gut microbial communities and virome have recently been identified to be correlated with T1D development (32, 33). For instance, butyrate- and acetate-producing bacteria have been associated with protection from spontaneous T1D in non-obese diabetic (NOD) mouse model of T1D (30). Bacterial metabolites, such as butyrate and acetate, can act on various inflammatory pathways to alter immune homeostasis (30, 34). Specifically, stimulation of the innate sensor, TLR5, induces the expression of butyrate receptor GPR43 on IECs enhancing T regulatory responses and modulating inflammation (35). In parallel, Gp43-deficient mice present with heightened inflammatory responses (36). In light of these findings, we believe that innate sensing is essential in priming the immune system during exposure to environmental antigens. As such, we hold that changes in innate immunity/sensing may indeed contribute to altered commensal microbiota as well as influence cellular permeability, all affecting the development of autoimmune disorders such as T1D. As such, Dr. Wallet’s research re-emphasizes the importance of innate signaling and commensal microorganisms and their respective influences on disease states.

Our lab has previously exhibited how differences of innate sensing in virus infections can trigger the onset of T1D autoimmunity. Polymorphisms in the interferon induced with helicase c domain 1 (*IFIH1*) gene have been strongly associated with T1D risk among patients (37). The virus sensor melanoma differentiation-associated protein 5 (MDA5) is expressed from *IFIH1* and recognizes ssRNA from viruses like CVB. NOD mice heterozygous for the MDA5 allele and thus expressing roughly half as much of the receptor as WT-NOD are protected from developing T1D following CVB4 infection whereas about 50% of the WT mice become autoimmune within 7 days post-infection (19). These MDA5 heterozygous mice produce a particular type 1 IFN response that appears to be protective for T1D and display an increased regulatory T-cell response. We have found that another ssRNA sensor, toll-like receptor 3 (TLR3), is critical for host defense to CVB4 and NOD mice deficient for TLR3 are highly susceptible to CVB4 infection (38). However, the mice that survive typically become diabetic, indicating that differences in TLR3 signaling may also contribute to T1D development (38). Thus, reduction of MDA5 but not TLR3 signaling is sufficient to down-regulate excessive inflammation that may subsidize autoimmunity. This work further exhibits how modulation of innate receptor activation alters the inflammatory profile and resulting adaptive response that induces or protects from T1D onset.

## Use of Tetramers for T1D Diagnostics and Targeted Therapeutics

Continuing the focus on immune mechanisms in disease pathology, assistant professor of Rheumatic and Autoimmune Disorders at the University of Minnesota and namesake of this symposium, Dr. Brian T. Fife, discussed the contribution of T cells to autoimmune pathology in T1D. The Fife group works on identifying and targeting autoreactive T cells in T1D using self-peptides and MHC II molecules conjugated in tetramer complexes (pMHCII tetramers) (39). This work aims to identify prediabetic individuals at risk of progressing to clinical disease, and to develop therapeutics against specific autoreactive T-cell subsets.

Using insulin peptide:MHCII tetramers, the Fife group demonstrated that the number of insulin-targeting CD4^+^ T cells in peripheral blood of T1D patients correlates with insulin autoantibody titers. These findings substantiate the feasibility of using pMHCII tetramers as a tool for early detection of autoreactive T cells in T1D. The Fife lab is currently developing an arsenal of tetramers against various diabetogenic targets, such as PD-1, which can be multiplexed to either eliminate or induce tolerance in autoreactive T cells (40). Theoretically, coupling the tetramers to toxins could selectively target particular subsets of autoreactive T cells for destruction, aiding in the re-establishment of self-tolerance. The Fife lab is also looking to use tetramers for antigen-specific-coupled cell tolerance, similar to insulin-coupled antigen-presenting cell (APC) therapy as previously published (41). Current efforts in the group are now focused on using pMHCII tetramers and T-cell receptor mimetic peptides as new T1D therapeutics that induces T-cell tolerance. The aim is for pMHCII tetrameric compounds to be used to specifically delete pathogenic T cells in patients.

The ability to identify and distinguish virus-specific and autoimmune-specific T cells is an especially advantageous process that has been universally utilized by many research groups. Tetramer technology has become an incredibly valuable and multifaceted biological tool which in this instance holds a two-sided benefit: not only may this technology be used for diagnostics but it also allows for therapeutics to be precisely delivered to the desired cells and the microenvironments in which they are harbored. Early detection of disease onset or predisposition of autoimmunity provides opportunity for early intervention to preserve beta cell mass and potentially even reverse presence of disease utilizing therapies such as those reviewed by Ludvigsson (42). Creating methods for heightened specificity and efficiency of tetramer identification for very distinct cells allows precise intervention and minimization of non-target destruction. Toxin-coupled tetramers provide opportunity to directly potentiate or eliminate the cell subsets responsible for self-reactivity. Recent work has shown that beta cells secrete neoantigens which further enhance the local T-cell response (43). Therefore, the Fife group is positioned to detail a comprehensive understanding and identification of what types of T1D-related self-antigens are produced, as well as which ones are critically targeted by pathogenic T cells allowing for the ability to provide intervention necessary for inducing antigen-specific T-cell tolerance.

## Recently Identified Th40 T Cells Promote Autoimmunity

Dr. David Wagner, from the University of Colorado, Denver Department of Medicine, discussed the role of CD40 in autoimmune inflammation. The recent discovery that the CD40 co-stimulatory molecule is expressed on T cells, not only APCs as previously thought, led to the hypothesis that CD40^+^CD4^+^ T cells, termed by Wagner as Th40 cells, could contribute to pathogenesis in autoimmunity. Impressively, Dr. Wagner examined the role of Th40 cells in both T1D and MS using murine models and patient data. In a CD40-reporter BDC2.5 T-cell transgenic murine model of T1D, hyperglycemia exacerbated CD40 expression in the pancreas (44). Adoptive transfer of CD40-depleted cell suspensions to NOD mice demonstrated that CD40-expressing cells are necessary and sufficient for the development of TID. Moreover, diabetogenic CD4^+^ T cells in the periphery of T1D patients expressed high levels of CD40, in contrast to T cells from healthy individuals. To examine the role of Th40 cells in MS, Dr. Wagner used the murine experimental autoimmune encephalomyelitis (EAE) model, an established immune-mediated model of MS. Adoptive transfer of splenic Th40 cells from EAE mice induced EAE in recipient naive mice, demonstrating the pathogenic capacity of Th40 cells (45). These results were then substantiated by the fact that the Th40 population is increased in MS patients irrespective of HLA haplotype, compared to age matched controls. Finally, Dr. Wagner’s team examined the therapeutic effects of inhibiting the interaction between CD40 and its ligand, CD154, using a KGYY15-blocking peptide. Blocking the CD40–CD154 interaction reversed hyperglycemia in new onset diabetic NOD mice and improved clinical scores in EAE mice. Importantly, the KGYY15 peptide bound to human T cells and reduced the ability of the T cells to produce IFN-γ. This research indicates that CD40 is a cellular pathologic marker in multiple autoimmune diseases and can be modulated for treatment of disease.

Overall, we feel Dr. Wagner’s research further substantiates not only the value of CD40 functionally, but also as a biomarker in viral and autoimmune pathology. Our lab has previously identified that APC expression of CD40 is a mechanism by which viral infection contributes to EAE by diminishing responding regulatory T-cell populations (46). Similarly, we propose that further examination of CD40 on both T cells and APCs in relation to viral infections in T1D onset is worthwhile. When exposed to neo-self-antigens in a transgenic OVA beta cell autoimmunity mouse model, Th40 cells lose the ability to express the immunoregulatory molecule, CTLA-4, as opposed to when in their naive state (47). Furthermore, transfer of antigen experienced CD40-expressing CD4^+^ T cells are able induce T1D in NOD.*scid* recipients (47). Increased CD40 expression leads to heightened secretion of inflammatory molecules and T-cell activation pushing immune homeostasis toward an inflammatory state instead of a tolerogenic one. Enteroviruses such as CVB have been strongly linked to T1D development; and blocking CD40 engagement in CVB3-induced inflammatory myocarditis has been shown to slow disease progress (48). Accordingly, CD40 may be contributing to an inflammatory state following infection that leads to autoimmunity and can potentially be used as a biomarker for pathogenic T cells. Determination of whether other viruses positively associated with T1D onset may be eliciting CD40-expressing immune cells could be important for understanding how these environmental pathogens are promoting development of autoimmunity.

## LAG-3-Mediated Immunoregulation Protects from T1D

Given that T-cell overamplification contributes to autoimmune disease etiology, attenuation of the effector functions, activation, and proliferation of diabetogenic T cells may impede the progression of T1D. Dr. Jon Piganelli, from the University of Pittsburgh’s Department of Immunology, examines homeostatic factors in relation to cell metabolic profiles to elucidate mechanisms of autoreactive T-cell development and persistence. Cleavage of the MHC II inhibitory receptor LAG-3 is a negative regulatory mechanism of immune cell activation; however, LAG-3 cleavage also prompts the metabolic transition from oxidative phosphorylation to glycolysis necessary for the activation and proliferation of T cells. Dr. Piganelli hypothesized that lack of LAG-3 results in T-cell overamplification, as fewer T cells are deleted during development and peripheral maturation, resulting in more aggressive autoimmunity. Indeed, LAG-3 knock-out NOD mice experience accelerated T-cell-mediated T1D (49). The group showed that LAG-3-deficient CD4^+^ T cells exhibit enhanced oxidative and glycolytic metabolism and increased mitochondrial biogenesis, supporting the hypothesis that overactive T cells lacking regulation contribute to T1D. Furthermore, inhibition of LAG-3 cleavage results in decreased T-cell proliferation and activation, as well as inhibition of metabolic switching in antigen-educated T cells. Ultimately, modification of LAG-3 is a potential therapy to prevent and treat effector T-cell-mediated autoimmune diseases such as T1D.

It is our view that virus infection shifts important checkpoints in cell regulation mechanisms in the development of T1D, by increasing local activation and stimulation. Rather than simply removing cell subsets involved in T1D pathogenesis, the work of Dr. Piganelli exhibits how immune cell factors may be targeted and can potentially be programmed to adopt a more tolerogenic state. Recent literature has shown that environment early on in life is important for incidence of T1D in NOD mice and exposure to a “diabetogenic environment” is sufficient to promote incidence (32). It was also determined that composition of certain bacterial pathobionts can induce immunophenotypic changes in mice weaned in this “diabetogenic environment” and harbor B cells in gut-related lymphoid organs which are intrinsically more easily activated by local stimulation (32). Environmental modification of homeostatic cell regulation pathways can necessarily predispose for increased microenvironment inflammation and cell activation that may be sufficient to induce autoreactivity. Therefore, determining pathways which may be safely targeted via drugs and therapeutics could point to effective disease treatments and prevention.

## Monocyte Expression of PTPN22 Potentiates T-Cell Recruitment and Activation

Type 1 diabetes is traditionally considered a T-cell-mediated disease and as such the bulk of T1D research is focused on the role of T cells (50–52). However, T cells require priming by myeloid professional APCs like dendritic cells (DCs) and macrophages. The upstream interaction between APCs and T cells is the focus of Dr. Mark A. Wallet’s research at the University of Florida, particularly the potential molecular mechanisms of DC regulation by the cytosolic phosphatase PTPN22. This protein, a known negative regulator of T-cell signaling, is expressed in DCs as well as monocytes and macrophages. Additionally, studies have shown that a coding variant polymorphism in human PTPN22 is associated with increased risk of T1D (53, 54). In mice, a similar polymorphism in PTPN22 leads to macrophage hyperactivation (55). To study the mechanisms of PTPN22 in regulation of human myeloid APCs, the Wallet team innovatively generated PTPN22-deficient monocytes from induced pluripotent stem cells where PTPN22 expression was ablated using CRISPR/Cas9-mediated gene targeting. When the PTPN22-deficient monocyte-derived DCs were treated with the TLR-stimulator zymosan, there was reduced expression of chemokines involved in recruitment of T cells, including CXCL10. This result indicated that PTPN22 may be involved in the recruitment of autoreactive T cells to the pancreas and enhance progression of T1D. However, lack of PTPN22 had no effect on amount of CD8^+^ T-cell proliferation or on downstream signaling following zymosan treatment. Meanwhile, the research remains to identify which receptor or receptors are driving CXCL10 and may be regulated by PTPN22. Overall, Dr. Wallet’s research shows that PTPN22 regulates the secretion of T-cell-recruiting chemokines by monocytes/DCs, shedding light on potential molecular mechanisms of T-cell priming and subsequent T1D pathogenesis.

We contend that the role of PTPN22 is incredibly complex and more work is necessary to determine how exactly this enzyme impacts T1D pathogenesis and whether genetic variation in *PTPN22* affects viral induction. APC interaction with T cells with regard to antigen presentation, stimulation, and chemical signaling can be detrimental for producing an autoreactive adaptive response to beta cells in T1D. PTPN22 has multiple roles in both the innate and adaptive immunity, affecting myeloid cell activation, T-cell proliferation and effector capacity, and secretion of type 1 interferons (56). The role of PTPN22 in various mouse models of T1D has been controversial (57). For instance, both diminishing and overexpressing PTPN22 were shown to reduce incidence of T1D in NOD mice (58, 59). Using a virus-mediated T1D mouse model (RIP-LCMV), PTPN22-deficient mice had increased incidence of T1D and resulted in an enhanced effector T-cell response to virus (60). Thus, it seems that PTPN22 deficiency positively affects virus-induced T1D but can protect in spontaneous disease. PTPN22 contributes to exhaustion of both CD4^+^ and CD8^+^ T lymphocytes and aids in establishment of chronic virus infections (61). We believe that chronic and persistence of certain types of viruses such as CVB are providing low-grade inflammation via interferon production in the pancreatic microenvironment that is triggering self-reactivity resulting in T1D. Dr. Wallet’s research further exemplifies how molecular mechanics for cell function can contribute to pathogenesis of T1D and immune disorder.

## Sepsis Complications in T2D is a Result of Impaired Bacterial Clearance

Dr. Matthew Delano, from the Department of Surgery at the University of Michigan, wrapped up the event by discussing T2D and susceptibility to infection. Dr. Delano approaches immunological diabetes research from the perspective of an acute trauma surgeon who has witnessed an abhorrent number of cases of sepsis among T2D patients. T2D, a disease caused by unresponsiveness to endogenous insulin, functions as an immunodeficiency that predisposes patients for infection (62). Dr. Delano hypothesized that defects in neutrophil function, previously linked to T2D-associated infections, directly contribute to bacterial persistence and death from sepsis (63). To examine the role of neutrophils in T2D, the Delano lab developed a novel diet-induced obesity (DIO) murine model with septic infection caused by cecal ligation and puncture. Neutrophils and monocytes in septic DIO mice failed to adequately phagocytose invading bacteria, resulting in increased bacterial persistence compared to their lean counterparts. The decreased phagocytic activity was caused in part by reduced ROS production. The group profiled the gene expression of neutrophils and monocytes and identified seven genes that were significantly and differentially expressed between septic DIO mice and lean controls. Most of these genes encoded receptors in pathways for phagocytosis, including particle recognition and engulfment. This work provided evidence that defects in neutrophil and monocyte function in T2D patients could account for persistence and/or susceptibility to sepsis following bacterial challenge. Dr. Delano is looking to target these identified genes to enhance phagocytosis and ROS production by neutrophils and monocytes as a therapeutic approach.

It is our perspective that understanding the contribution of viral infection in T2D is paramount to developing immunotherapies. Sepsis causes significant changes in nearly every type of innate and adaptive immune cells which persist well after septic acute phase and efforts are being made to develop immunotherapies to combat this dysfunction (64). By considering T2D as an immunodeficiency that predisposes patients to secondary infections due to defects in neutrophil function, Dr. Delano’s work reestablishes the importance of investigating innate immune processes in disease development and progression, simultaneously proposing a parallel to T1D pathogenesis and defects in innate sensing. Overall, this research emphasizes the necessity of examining other pathogenic exposures and the extent of their effect on disease priming, especially within the context of cross-reactivities and heterogeneous disease presentations.

## Conclusion

The multidisciplinary FIFE mini-symposium brought together young researchers from across North America investigating various interconnected contributors to TID onset and progression and put forth multiple concepts for further examination. Common themes of interest included autoreactive T-cell expansion and persistence, T-cell activity alteration and loss of homeostatic mechanisms, and environmental exposures, including infections and the microbiota. Immunotherapeutic targets, and methods for delivering treatments to the pancreatic microenvironment or specifying them to specific autoreactive subsets, were proposed. Moving forward, the first 2016 FIFE symposium provided a foundation from which investigators can exchange ideas and form collaborations to advance diabetes research. It is our goal that future FIFE collaborative efforts be planned to provide a positive environment and forum for communication and idea generation with a goal to aid in the prevention and cure of T1D. Next year, we will see you in Gainesville, Florida!

## Author Contributions

IM helped write sections and edited and organized the manuscript. ZM added perspective to all the sections. ZM, VJ-B, and JA wrote sections and edited. MH edited and oversaw the manuscript.

## Conflict of Interest Statement

The authors declare that the research was conducted in the absence of any commercial or financial relationships that could be construed as a potential conflict of interest.
